# Surface Characteristics and Biological Evaluation of Si-DLC Coatings Fabricated Using Magnetron Sputtering Method on Ti6Al7Nb Substrate

**DOI:** 10.3390/nano9060812

**Published:** 2019-05-29

**Authors:** Dorota Bociaga, Anna Sobczyk-Guzenda, Piotr Komorowski, Jacek Balcerzak, Krzysztof Jastrzebski, Karolina Przybyszewska, Anna Kaczmarek

**Affiliations:** 1 Faculty of Mechanical Engineering, Institute of Materials Science and Engineering, Lodz University of Technology, 1/15 Stefanowskiego St., 90-924 Lodz, Poland; anna.sobczyk-guzenda@p.lodz.pl (A.S.-G.); piotr.komorowski@p.lodz.pl (P.K.); kj.drakkainen@gmail.com (K.J.); przybyszewska.karolina94@gmail.com (K.P.); anna.m.kaczmarek90@gmail.com (A.K.); 2 Bionanopark Ltd., Molecular and Nanostructural Biophysics Laboratory, 114/116 Dubois St., 93-465 Lodz, Poland; 3 Faculty of Process and Environmental Engineering, Department of Molecular Engineering, Lodz University of Technology, 213 Wolczanska St., 90-924 Lodz, Poland; jacek.balcerzak@p.lodz.pl

**Keywords:** DLC bio-functionality, silicon doping, diffusion barrier, biocompatibility, proliferation improvement, endothelial cells

## Abstract

Diamond-like carbon (DLC) coatings are well known as protective coatings for biomedical applications. Furthermore, the incorporation of different elements, such as silicon (Si), in the carbon matrix changes the bio-functionality of the DLC coatings. This has also been proven by the results obtained in this work. The Si-DLC coatings were deposited on the Ti6Al7Nb alloy, which is commonly used in clinical practice, using the magnetron sputtering method. According to the X-ray photoelectron spectroscopy (XPS) analysis, the content of silicon in the examined coatings varied from ~2 at.% up to ~22 at.%. Since the surface characteristics are key factors influencing the cell response, the results of the cells’ proliferation and viability assays (live/dead and XTT (colorimetric assays using tetrazolium salt)) were correlated with the surface properties. The surface free energy (SFE) measurements, Fourier transform infrared spectroscopy (FTIR), and X-ray photoelectron spectroscopy (XPS) analysis demonstrated that the polarity and wettability of the surfaces examined increase with increasing Si concentration, and therefore the adhesion and proliferation of cells was enhanced. The results obtained revealed that the biocompatibility of Si-doped DLC coatings, regardless of the Si content, remains at a very high level (the observed viability of endothelial cells is above 70%).

## 1. Introduction

Implants are introduced into the body in order to recreate different mechanical and biological functions, and therefore improve the quality of human life or even save it. Since implants remain in constant contact with the living tissues and body fluids, they must fulfill strict requirements concerning their mechanical and biological properties. Currently, the most frequently implanted biomaterials are metals, which may be further classified into three main groups, i.e., stainless steels, titanium-based alloys, and cobalt-based alloys [1,2,3]. Metallic biomaterials possess high strength, resistance to fracture, as well as good corrosion resistance. However, biomaterials, in order to be safely inserted into the living organism, should also exhibit high biocompatibility, which means they must not cause any adverse tissue reactions such as acute and chronic inflammation or irritation of the surrounding tissues. Despite good mechanical properties, even metals with good corrosion resistance do not show full chemical stability in the highly aggressive environment of the human body. Therefore, metallic biomaterials may release harmful degradation products, which includes metal ions that can cause a negative biological response. The released degradation products not only accumulate in the surrounding tissues and organs, but also induce inflammation [4] and allergic reactions [5]. As a consequence, post-implantation infection may occur and lead to the revision or removal of the implant [6,7,8,9]. 

Hence, today, growing attention has been given to the modification of the surface of metallic biomaterials in order to enhance their properties, also in the context of a biological response [10]. One of the most commonly investigated solutions is the use of diamond-like carbon (DLC) coatings [11,12]. According to the numerous studies carried out by different scientific groups, DLC films exhibit high wear resistance [13], very good physiochemical properties [14], superior corrosion resistance [15], as well as excellent bio- and hemocompatibility [16,17,18,19,20,21]. Therefore, diamond-like carbon coatings can act as protecting coatings for implants and reduce the risk of adverse tissue reactions due to the implantation [22].

Moreover, the incorporation of different elements into the carbon matrix may result in even further improvement of certain properties of DLC films. Depending on the dopant used, different features may be achieved, including varying bio-functionality which determines the potential biomedical applications of the doped diamond-like carbon films. However, according to the literature, the biological response towards the doped DLC coatings depends not only on the element used, but also on the physiochemical properties of the surface. Those are in turn controlled by the synthesis method, different parameters of the deposition process, the form and amount of the incorporated dopant, as well as the substrate itself. 

One of the most vastly investigated elements applied as a dopant for DLC coatings is silicon (Si), which according to the literature improves hemocompatibility, cell proliferation, and antibacterial resistance [23,24,25,26,27]. De Scheerder et al. also proved that the inflammatory reactions may be inhibited by the incorporation of SiO_x_ in the diamond-like carbon films [28]. Furthermore, the addition of Si into the DLC matrix enhances its chemical, mechanical, as well as tribological characteristics, causing the increase of hardness along with the reduction of the friction coefficient and residual stress [29,30,31,32]. As a result, the Si-incorporated carbon films may serve as a barrier which effectively prevents the diffusion of metal ions from the substrate, and therefore improves the biocompatibility of metallic biomaterials. 

Despite numerous studies concerning the Si-DLC coatings, there are only a few reports concerning the evaluation of Si-DLC coatings deposited on titanium-based alloys using the magnetron sputtering technique. Therefore, the aim of this work was to perform a complex study concerning the physiochemical and biological characteristics of Si-doped DLC coatings deposited on Ti6Al7Nb substrates via co-sputtering of silicon and graphite targets.

## 2. Materials and Methods 

### 2.1. Deposition of the Diamond-Like Carbon (DLC) DLC and Si-DLC Coatings

The DLC and Si-DLC coatings were both fabricated on Ti6Al7Nb substrates (Bibus Metals Sp. Z o.o. [Ltd.], Dabrowa, Poland). The applied discoidal samples had a diameter of 16 mm and a thickness of 6 mm. Before the deposition process, all substrates were ground and polished using the OP-S silica suspension (StruersApS). This was followed by ultrasonic cleaning in acetone for 10 min.

For the deposition of the examined DLC and Si-DLC films the multi-target magnetron sputtering system (PREVAC Sp. z o.o. [Ltd.], Rogow, Poland) was used. The apparatus consisted of a chamber with peripheral accessories, a control cabinet, and a computer enabling remote control of individual components. The chamber was equipped with three magnetron guns ION’X® (Thin Film Consulting, Grafenberg, Germany) with individual cooling systems. Two of them worked in a constant current mode, Pinnacle series power supplies (Advanced Energy, Fort Collins, CO, USA), while the third one was operated using a high frequency RF generator, Cesar RF Power Generator (Advanced Energy, Fort Collins, CO, USA). The working pressure was ensured by a pump system consisting of a SCROLLVAC CS 30D primary pump (Leybold GmbH, Köln, Germany) and Turbovac SL700 turbo pump (Leybold GmbH, Köln, Germany). The rotary pump was used both to obtain the initial vacuum in the working chamber and to preserve the low operating pressure of the turbo pump. The system was equipped with two graphite (DC—direct current) and one silicon (RF—radio frequency) sputter targets (Kurt J. Lesker Company, Jefferson Hills, PA, USA) with a purity of 99.999%. In order to assure the uniform thickness of the coatings the samples were placed on a rotary table moving at a constant velocity of 0.33 rpm. Prior to the deposition, the plasma etching in argon atmosphere was carried out for 30 minutes at the bias of −700 V and pressure equal to 1 Pa. The input power of both graphite targets during the synthesis process of the DLC and Si-DLC coatings was 200 W each, while the pressure value was kept constant at 0.6 Pa with the Ar flow of 10 sccm. The silicon was introduced by co-sputtering of the silicon target with the input power varying from 0 to 80 W in order to achieve different Si concentrations. The deposition time was equal to 60 min. 

### 2.2. Surface Morphology by Scanning Electron Microscopy (SEM)

The surface morphology of the deposited DLC and Si-DLC coatings was analyzed using the scanning electron microscope JSM-6610LV (JEOL). The secondary (SE) electron imaging mode was applied in order to obtain topographical contrast, while the backscattered (BSE) electrons imaging mode was used for compositional contrast. The applied accelerating voltage was equal to 20 kV. All of the performed SEM observations were carried out under high vacuum. 

### 2.3. Chemical Composition and Bonding by X-ray Photoelectron Spectroscopy (XPS)

The surface chemical composition of the examined DLC and Si-DLC films was investigated by X-ray photoelectron spectroscopy. For this purpose, the Kratos AXIS Ultra spectrometer was used. The system was equipped with a monochromatic Al Kα X-ray source with an excitation energy of 1486.6 eV. High resolution measurements were performed with the power of the anode equal to 150 W and the pass energy of the hemispherical electron energy analyzer set to 20 eV. The XPS spectra were collected from the analysis areas of 300 µm × 700 µm. The obtained data were used to establish a quantitative elemental composition of the coatings. Moreover, the atomic bonds between the elements were studied in detail.

### 2.4. Chemical Structure by Fourier Transform Infrared Spectroscopy (FTIR)

The analysis of the chemical structure of the deposited DLC and Si-DLC coatings was carried out using Fourier transform infrared spectroscopy (FTIR). Investigations were performed using a Nicolet iS50 spectrophotometer (Thermo Scientific) operated in the absorbance mode in the range of 1700–500 cm^−1^. The resolution of spectral measurements was equal to 1 cm^−1^ and a single measurement cycle consisted of 120 scans. 

### 2.5. Surface Free Energy (SFE) and Wettability

The wettability of the DLC and Si-DLC coatings was measured using the sessile drop technique for two liquids of different polarity and known surface tension, i.e., distilled water and diiodomethan (Sigma Aldrich). The FM40 Easy Drop system with Drop Shape Analysis software (Krüss GmbH, Hamburg, Germany) was applied for that purpose. Prior to the measurements, steam sterilization of all of the examined samples was conducted in order to reflect the state of the surface in contact with the biological material.

The wettability measurements were followed by the assessment of the surface free energy. This was based on the Owens-Wendt theoretical model, and accordingly, the SFE of a solid consists of two components, dispersive and polar [33,34]. The following equations were used for the determination of the SFE values:

γL × (1 + cosθ)/2 = (γSd × γLd)^0.5 + (γSp × γLp)^0.5(1)

γS = γSd + γSp (2)

where, γL—liquid’s surface free energy; γS—solid’s free energy; γSd, γSp—dispersive (d) and polar (p) component of the surface energy γS; γLd, γLp—dispersive (d) and polar (p) component of the surface energy γL; θ—contact angle.

### 2.6. Endothelial Cells’ Viability and Proliferation

The biocompatibility of the DLC and Si-DLC films was evaluated using human endothelial cell line, EA.hy926, (ATCC - American Type Culture Collection). All of the samples were ultrasonically cleaned in ethanol and then in ultrapure water (0.055 µS/cm) for 15 min. Next, steam sterilization (121 °C, 15 min) was performed using the autoclave (J.P. Selecta Autoclave 401731).

Afterwards, the endothelial cells were seeded onto the surface of the examined samples at a density of 6 × 10^4^ cells per well. The cultures were carried out in DMEM medium (Biowest, Nuaillé, France) for 48 h in standard conditions (i.e. 37 °C) and humidified atmosphere of 5% CO_2_ in air. The cells with no contact with any biomaterial were used as a control, as well as the Ti6Al7Nb substrates.

#### 2.6.1. Live/Dead Assay

The proliferation and viability of the endothelial cells on the surface of the examined samples were evaluated by means of live/dead assay. The IN Cell Analyzer 2000 (GE Healthcare, Chicago, IL, USA) automated microscope was used in order to visualize live and dead cells. Prior to the microscopic observations, the samples were incubated in Hank’s Balanced Salt Solution containing fluorescent dyes at room temperature for 15 min. The applied mixture of fluorescent dyes included: 5 µg/mL of Hoechst 33342 (Molecular Probes, Eugene, OR, USA), 1–5 µM calcein-AM (Santa Cruz Biotechnology, Dallas, TX, USA), and 1 µg/mL of propidium iodide (Molecular Probes). The analysis of the obtained series of microscopic images was performed using the IN Cell Analyzer software (GE Healthcare, Chicago, IL, USA). The classification of EA.hy926 cells into two subpopulations, (i.e. live and dead) was based on the observed fluorescent signal. The cells stained with the green-fluorescent calcein-AM (retained in the cytoplasm) were labelled as live, while cells stained with the red-fluorescent propidium iodide were counted as dead. The percentage of live and dead cells on the surface allowed assessment of its cytotoxicity. The proliferation of the cells, understood as the total cells’ count, was evaluated on the basis of the blue-fluorescent signal coming from cells’ nuclei stained with Hoechst 33342 dye which intercalates to the DNA.

#### 2.6.2. XTT Assay

The XTT assay, based on the mitochondrial activity of the cells, was used in order to assess the viability of cells which were in, both, direct and indirect contact with the surface of the deposited DLC and Si-DLC coatings. For that purpose, the medium was removed after 48 h of culture and the XTT solution (XTT Cell Viability Assay Kit, Biotium Inc., Fremont, CA, USA) was added in accordance with the procedure described by the manufacturer. The samples were then incubated in the XTT solution for 4 h in standard conditions (37 °C, humidified atmosphere of 5% CO_2_ in air). After that, the absorbance was measured using a Multiskan GO microplate spectrophotometer (Thermo Fisher Scientific, Waltham, MA, USA) at two different wavelengths, i.e., 450 nm and 620 nm (reference). The following formula was applied to calculate the viability of the endothelial cells:

Viability [%] = (A/Ac) × 100% (3)

where, A—absorbance measured for the investigated sample and Ac—absorbance measured for the control (cells with no contact with any biomaterial at all).

## 3. Results and Discussion

### 3.1. Surface Morphology by Scanning Electron Microscopy (SEM)

**Figure 1:**
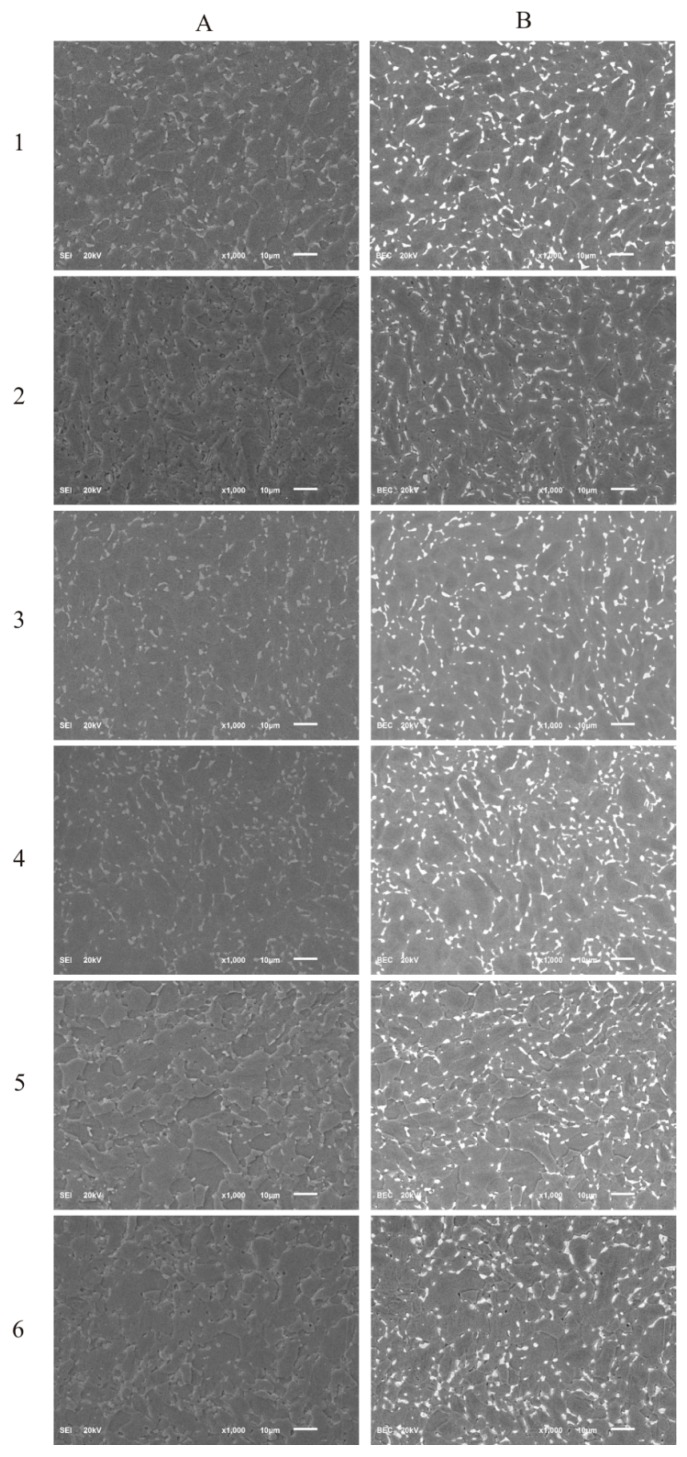
The scanning electron microscopy (SEM) images of the deposited coatings: (**A**) secondary electron imaging; (**B**) backscattered electron imaging, (**1**) diamond-like carbon (DLC), (**2**) Si-DLC 0, (**3**) Si-DLC 1, (**4**) Si-DLC 2, (**5**) Si-DLC 3, (**6**) Si-DLC 4.

The SEM images of the surface morphology of the DLC and Si-DLC films are presented in Figure 1. No significant changes in the morphology of the examined coatings due to the addition of silicon were observed in both the topographic (Figure 1A) as well as the compositional (Figure 1B) images. The SEM examination revealed that the surface of the fabricated coatings was smooth and uniform without any defects or delamination, which proves the high quality of the obtained magnetron sputtered films. Furthermore, no silicon conglomerates were observed. However, due to the very low thickness of the deposited coatings (~250 nm), the contrast arising from the two-phase Ti6Al7Nb alloy may be observed. 

### 3.2. Chemical Composition and Bonding by X-ray Photoelectron Spectroscopy (XPS)

**Figure 2:**
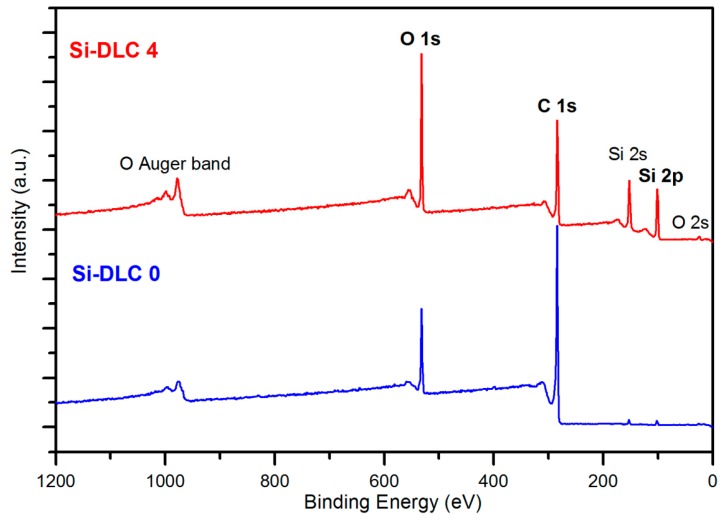
The X-ray photoelectron spectroscopy (XPS) wide scans of the surface of the Si-DLC 0 and Si-DLC 4 samples.

The comparative XPS wide scans of exemplary surfaces of samples, Si-DLC 0 and Si-DLC 4, are depicted in the Figure 2. The spectra revealed that the examined Si-DLC coatings are only composed of oxygen (O 1s and O 2s band), carbon (C 1s band), and silicon (Si 2s and Si 2p band), without any impurities.

The results of the quantitative XPS analyses of all of the samples’ surfaces are presented in the Table 1. Data shown confirms that with increasing magnetron sputtering power, the relative atomic content of silicon also increases. 

**Figure 3:**
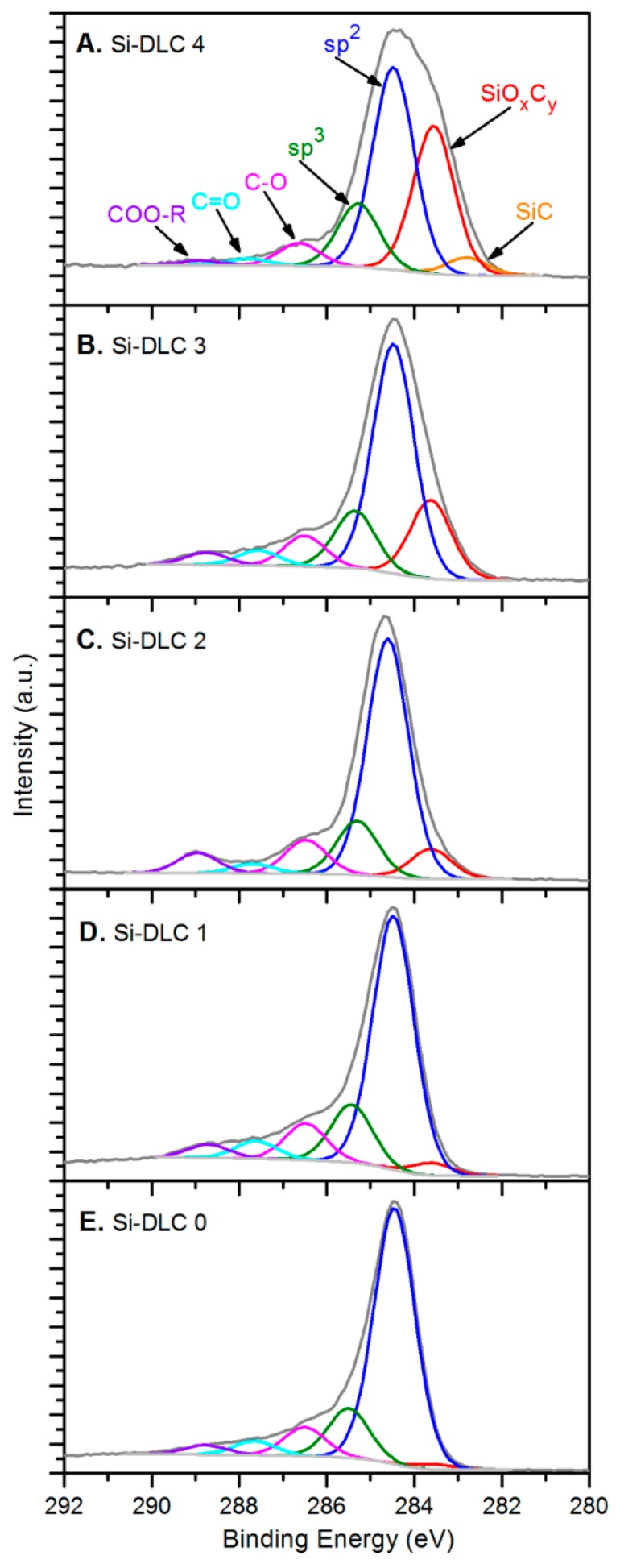
The deconvolution of the C 1s band for the deposited Si-DLC coatings: (**A**) Si-DLC 4, (**B**) Si-DLC 3, (**C**) Si-DLC 2, (**D**) Si-DLC 1, (**E**) Si-DLC 0.

In terms of the chemical structure of the deposited films, high resolution XPS analysis of oxygen O 1s, carbon C 1s, and silicon Si 2p bands was conducted. Figure 3 presents a C 1s band for each of the Si-DLC coatings divided into components representing specific atomic bonds.

The spectra of the samples from the Si-DLC 3 to the Si-DLC 0 (Figure 3B–E) were deconvoluted, according to the literature [35,36], into six components assigned to: (SiO_x_C_y_) silicon oxycarbides (at 283.60 eV), sp^2^ hybridized carbon (at 284.50 eV), sp^3^ hybridized carbon (at 285.30–285.50 eV), C-O bonds (at 286.50 eV), C = O bonds (at 287.60–287.80 eV), and ester bonds COO-R (at 288.70–288.90 eV). Additionally, for the Si- DLC 4 film, also the Si-C bond was distinguished in the position of 282.80 eV (Figure 3A), which was reported in the literature [36]. The relative content of the abovementioned components, identified in carbon C 1s band, is presented in the Table 2 for every investigated sample.

**Figure 4:**
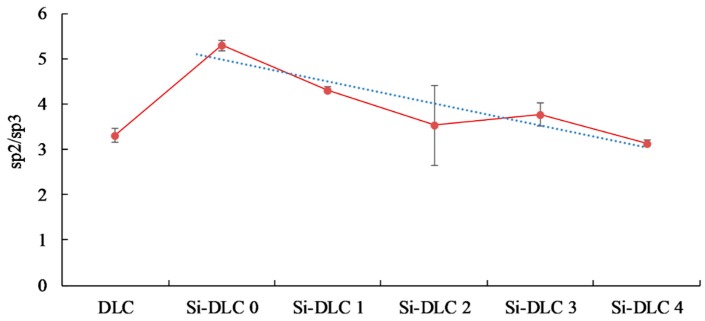
The sp^2^/sp^3^ bonding ratio of the deposited DLC and Si-DLC coatings.

The analysis of the data presented in Table 2 and the comparison of the C 1s spectra depicted in Figure 3 led to the conclusion that the increase of the magnetron sputtering power results in the significant evolution of silicon oxycarbide and eventually the formation of silicon carbide bonds. Additionally, a slight decrease of the sp^2^/sp^3^ hybridization ratio may be also observed as a result of the increasing sputtering power and higher Si content (Figure 4).

Decomposition of the O 1s band, presented in Table 3, confirmed the oxycarbide bonds relative content depended on the magnetron sputtering power and the Si content in the structure of the Si-DLC films.

### 3.3. Chemical Structure by Fourier Transform Infrared Spectroscopy (FTIR)

**Figure 5:**
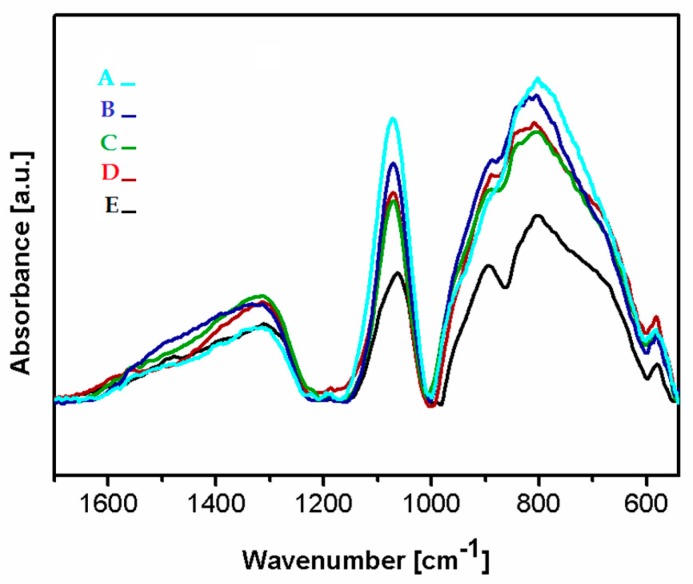
The Fourier transform infrared spectroscopy (FTIR) spectra of the deposited coatings Si-DLC coatings: (**A**) Si-DLC 4, (**B**) Si-DLC 3, (**C**) Si-DLC 2, (**D**) Si-DLC 1, (**E**) Si-DLC 0.

Figure 5 presents the FTIR spectra of the deposited Si-DLC coatings in the range of 1700–500 cm^−1^. In the analyzed region, three wide bands can be distinguished at 1650–1230 cm^−1^, 1160–1000 cm^−1^, and 1000–540 cm^−1^. In the range of 1650–1230 cm^−1^ the maxima corresponding to the C = O bonds (1580 cm^−1^), the – CH_3_ and/or –OH groups (1412–1370 cm^−1^), the SiOCOCH_3_ groups (1350 cm^−1^), as well as the C = O (1305 cm^−1^) can be observed [37,38,39]. The presence of the – CH_3_ groups in the deposited coatings may be due to the contamination of the graphite targets used as a source of carbon during the deposition process. The C = O bonds are most probably present at the surface of the deposited coatings due to the oxidation of the surface after the deposition process. According to Ong et al. [40], the absorbance of the oxygen atoms from the atmosphere may occur as a result of the unfused bonds present on the surface of the deposited coatings.

**Figure 6:**
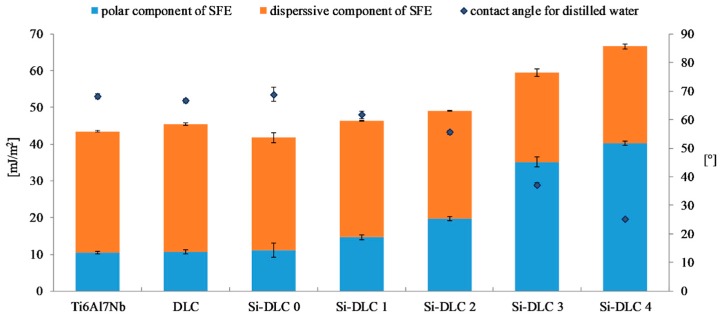
The surface free energy and wettability of the deposited DLC and Si-DLC coatings after sterilization.

In the range of 1160–1000 cm^−1^, vibrations of bonds belonging to the Si-O groups with a high-intensity peak at 1080 cm^−1^ are noticed. This maximum may be assigned to the stretching modes of the Si-O-C bonds [39,41,42]. Thus, in order to evaluate the difference in the content of the Si-O bonds in the deposited films, the peak at 1080 cm^−1^ was used. The lowest intensity of this peak was revealed for the Si-DLC 0 coatings with the lowest content of Si. With the increasing concentration of Si in the examined coatings, the intensity of the analyzed peak also increased. The Si-O bond may be responsible for the change in the surface wettability (Figure 6). With the increasing content of the Si-O bonds, the surface polarity increases and, as a consequence, also the surface wettability is raised, which may influence the biological response. 

In the range of 1000–500 cm^−1^, the peaks corresponding to the stretching modes of the Si-CH_3_ bonds at (905 cm^−1^) and the Si-O-C groups at 830 cm^−1^, as well as in the non-hydrogenated Si-C groups (800 cm^−1^) are observed. Moreover, the peaks originating from bending vibrations of the C = O bonds are noticed at 670 and 568 cm^−1^, respectively [39,41,42]. 

It is observed that as the Si concentration in the investigated coatings increases, the amount of the Si-CH_3_ bonds also increases, except for the Si-DLC 4 sample. In this coating, the content of the Si-CH_3_ bonds is reduced, but the amount of the Si-C bonds increases. The peak corresponding to the vibrations of the Si-C bonds in the spectrum of the coating with the highest content of Si (Si-DLC 4) is also shifted towards the lower wave numbers, which may be connected to the fact that silicon in the structure of this coating is mostly bonded to the non-hydrogenated carbon. The increase in the nonpolar Si-C bonds does not result in the increase in the dispersive component of the SFE. On the contrary, the opposite effect is observed (Figure 6) and the increasing content of Si resulted in the higher surface polarity and wettability. This may be due to the fact that the Si-O and the C=O bonds, which are polar in nature, are localized mainly at the surface of the fabricated films which are directly responsible for the increasing polar component of the SFE with the increasing content of Si. 

### 3.4. Surface Free Energy (SFE) and Wettability

Figure 6 presents the results of the surface free energy and wettability assessment of the deposited coatings. It is observed that the wettability of the Ti6Al7Nb substrate, DLC, and Si-DLC 0 coatings is similar. However, for the higher concentrations of Si, the wettability of the surface significantly increases. At the same time, it must be noted that all the examined surfaces are hydrophilic. The higher the content of Si, the higher the wettability of the surface. The results obtained confirmed the findings presented by Ong et al. [40] and Okpalugo et al. [43] who found that the polar component of the surface free energy is higher as Si content increases, especially when it exceeds 16 at.%. Similar results were also reported in our previous work [27].

The changes in the SFE and wettability are explained by the differences in the chemical structure of the deposited coatings. The O 1s region of the XPS spectra of the investigated films is deconvoluted into the following components: C = O, Si-O-C, and SiO_x_ bonds. For higher magnetron sputtering powers, the content of the SiO_x_ bonds decreases despite the increasing concentration of Si. Moreover, the contribution of the C = O bonds is reduced from 10.9%, for the Si-DLC 0 coatings with 1.83 at.%. of Si, to zero, for the Si-DLC 4 coating containing 22.15 at.%. of Si. At the same time, the content of the Si-O-C groups increases which is especially significant for the Si-DLC 4 coating with the highest amount of Si. The most notable growth of the surface polarity is observed for the Si-DLC 3 and the Si-DLC 4 coatings, as seen in the Figure 6. This may be explained by the fact that in the Si-O-C groups two different bonds are present, i.e., the covalent C-O bond and the ionic Si-O bond. Due to the fact that the ionic bond is stronger than the covalent bond, the breakdown of the C-O bonds is much more possible. The newly created unfused bonds may be saturated with water vapour. As a result, the C-OH and Si-OH groups are formed, which causes the increase of hydrophilicity of the surface. In addition, in the case of the Si-DLC 4 sample, the CH_3_ groups (responsible for the dispersion component of the SFE) disappear, which also leads to the increase of the surface wettability.

### 3.5. Endothelial Cells’ Viability and Proliferation

In general, cells show good spreading, proliferation, and differentiation on hydrophilic surfaces. Nevertheless, the major factor determining the nature of the cells’ interaction with biomaterials is the composition and conformation of the proteins adsorbed on the surface. Due to the fact that the adhesion of the cells to the surface of the material requires a series of cytoplasmic, transmembrane, and extracellular proteins which assemble into the stable contact sites [44], the adsorption of serum and extracellular matrix proteins is likely affecting the adhesion and behavior of cells [45]. Therefore, the observed difference in the proliferation and adhesion of endothelial cells may be caused by the difference in the absorption of proteins responsible for the cell colonization process. This is especially important in the case of proteins involved in the formation of the extracellular matrix (ECM), such as proteoglycans and glycoproteins, i.e., fibronectin, laminin, and collagen. In addition to the amount of proteins adsorbed on the surface, their biological activity, which is connected, for example, with their conformation, may be changed [46,47]. Moreover, the size of the biological molecules and the time of contact with the biomaterial’s surface can determine the tissue–biomaterial interaction [48].

**Figure 7:**
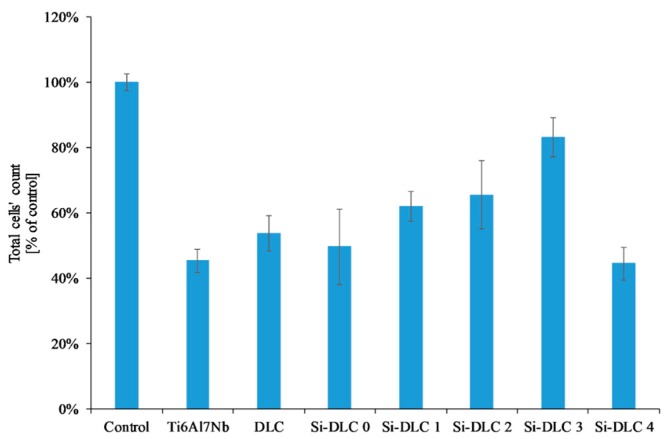
Endothelial cells’ proliferation on the surface of the deposited Si-DLC coatings (live/dead assay).

The adsorption of proteins responsible for the cell colonization and their activity may be affected by the physiochemical properties of the surface, i.e., the surface free energy and the associated surface wettability, as well as the charge and chemical composition of the biomaterial’s surface. According to the literature, the higher surface wettability results in better adhesion and proliferation of the eukaryotic cells [46,49,50,51] This was confirmed by the results obtained. The life/dead assay showed that with the increasing content of Si, the cells’ viability on the surface of the deposited coatings is enhanced as a result of the increased surface hydrophilicity. The biocompatibility evaluation of the deposited Si-DLC coatings demonstrated slightly enhanced proliferation of the endothelial (EA.hy926) cells, and adhesion on the surface of the Si-free DLC coating as compared with the Ti6Al7Nb substrate (Figure 7). Moreover, the incorporation of Si further improves the viability of endothelial cells on the surface of the Si-DLC coatings, especially for the Si-DLC 3 sample with 14.34 at.%. of Si. The higher the sputtering power and the resulting Si content, the more hydrophilic surface is obtained. This is due to the higher content of the Si-O bonds, which increase the polar component of the SFE and change the surface wettability. The higher the concentration of Si, the higher the wettability of the surface observed. In the case of the Si-DLC 4 coating where the amount of Si was above 16 at.%., the increase in the polar component of the SFE and wettability of the surface was still observed [40,43], but their effect on the behavior of the cells was suppressed and the reduction of proliferation of the cells occurred. Nevertheless, the biocompatibility of Si-doped DLC coatings, regardless of the Si content, remains at a very high level.

Another important factor influencing the cell colonization of the surface is the structure of the DLC coatings, i.e., the sp^2^/sp^3^ ratio [52]. According to the literature, the Si element increases the sp^3^ content. As the silicon content increases, the sp^2^/sp^3^ ratio decreases and as a result the proliferation of the cells is enhanced. According to T.T Liao el at. [50], the sp^2^ bonding, characteristic of the graphite phase, results in lower adsorption of proteins and cells on the surface, while the sp^3^ bonding, typical for the diamond phase, improves cell colonization. This is explained by the presence of free electrons on the surface of the diamond-like carbon coatings (the sp^2^ phase) which suppress the adhesion of proteins and cells. A similar tendency was revealed for the examined coatings. With an increasing amount of Si, the content of the sp^3^ also increased (Table 2), which, in turn, resulted in a higher proliferation of the endothelial cells. The only exception was the Si-DLC 4 film with the highest concentration of Si (22.15 at.%.). Despite the highest surface wettability and the lowest content of the sp^2^, the proliferation of cells on the surface of the Si-DLC 4 coatings was lowered as compared with other Si-DLC coatings (similar to the Ti6Al7Nb sample without the DLC coating). This may be due to the fact that the content of Si in the Si-DLC 4 coating is too high and it affects the process of cells’ division. The critical concentration of Si may be different for different cell lines and it may be associated with cellular adaptation [48,53]. Moreover, according to some authors, the high hydrophilicity of surface may inhibit the adhesion of cells due to the preferential adsorption of water molecules [54]. In this case, it can also be connected to the influence of surface charge on the behavior of cells [55]. According to Thevenot et al., the presence of negative charges may facilitate the adsorption of proteins promoting adhesion of the cells [44]. Moreover, Keselowsky et al. reported that surfaces with differently charged functional groups (–CH_3_, –OH, –COOH, and –NH_2_ groups) positively influenced the adsorption of fibrynogen as well as direct integrin binding and specificity [55]. In addition, Schmidt et al. pointed out that the functional groups such as –CH_3_, –OH, –COOH affect the proteins responsible for cells’ behavior [56]. In the case of the sample Si-DLC 4, the content of the Si-CH_3_ bonds is reduced, while the amount of the SiO_x_C_y_ increases significantly. According to the Lagonegro et al. [57] the silicon oxycarbide reduces the cells’ proliferation ability. Therefore, for the amount of Si above 20 at.%., the chemical and structural changes in the Si-DLC coatings caused the deterioration of biocompatibility. 

**Figure 8:**
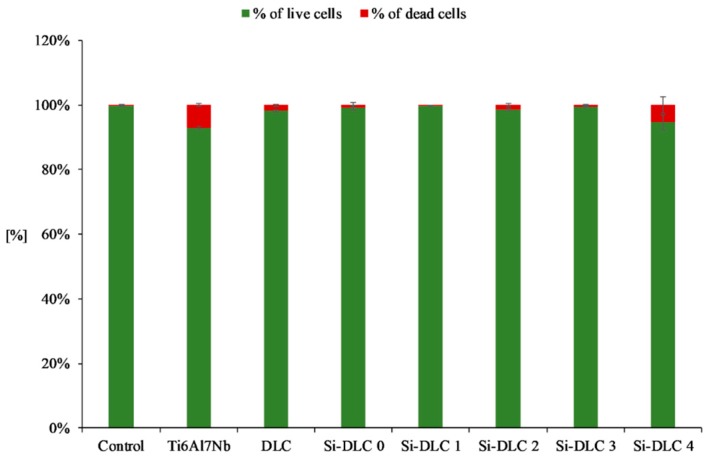
Cytotoxicity of the deposited Si-DLC coatings towards the endothelial cells (live/dead assay).

Furthermore, the live/dead assay (Figure 8) demonstrated that no significant increase in the number of dead cells for any of the examined samples was observed. This proves that none of the deposited coatings exhibit cytotoxicity towards the endothelial cells.

**Figure 9:**
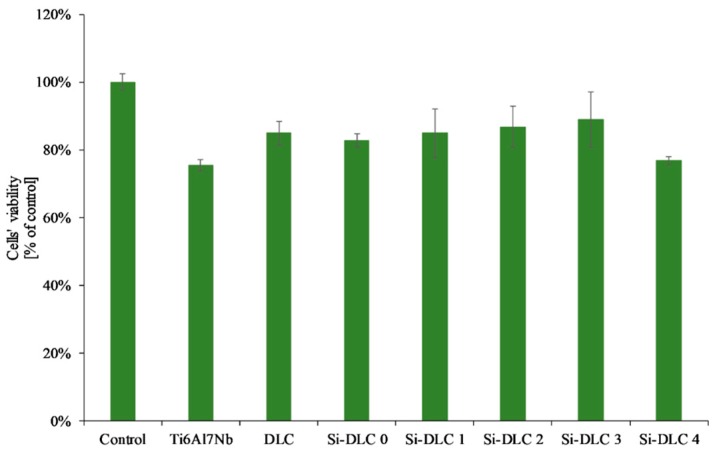
Endothelial cells’ viability in direct and indirect contact with the deposited Si-DLC coatings (XTT assay).

As far as the XTT assay was concerned (Figure 9), it revealed that the Si incorporation into the DLC matrix had no significant influence on viability of EA.hy926 cells in indirect contact with the investigated surfaces. The overall change in the mitochondrial activity of endothelial cells in contact with the deposited Si-DLC coatings is negligible. Only a slight raise in the mean value of cells’ viability may be noticed with the increasing Si content up to 14.34 at.%., followed by a small decrease for the coating with 22.15 at.%. of Si. This may be explained by the changes in the cells’ proliferation and viability on the surface of the fabricated coatings as indicated by the results of the live/dead assay.

To sum up, according to the ISO 10993-5, a biomaterial may be considered biocompatible if the cells’ viability is higher than 70% [58] and, according to the results presented in this work, this criterion was fulfilled by all of the analyzed Si-DLC coatings, as the observed cells’ viability was above 70%. 

## 4. Conclusions

The results presented in this work showed that the magnetron sputtering is an effective method to produce homogenous and biocompatible Si-doped DLC coatings. The use of varying sputtering powers allows a different content of silicon in the coatings to be obtained, which affects both the chemical composition and structure, and therefore the surface properties and biological response. The addition of silicon to the DLC coating deposited on the Ti6Al7Nb alloy has a very positive effect on the proliferation and viability of endothelial cells. 

The higher the concentration of Si, the higher the wettability of the surface observed. The increase of the polar component of the SFE and wettability of the coatings with the highest amount of Si is connected with the high content of the Si-O bonds. Additionally, for the Si-DLC 4 sample the lower content of the –CH_3_ groups (responsible for the dispersive component of the SFE) is observed and the C-OH and Si-OH groups are formed which causes the increase of the polar component. According to the literature, the increase in the surface wettability promotes the cells’ proliferation. Our research has shown that this relationship exists, but only to a certain level. A change in the biological response observed for the Si-DLC 4 coating may indicate that Si is tolerated by the endothelial cells up to a limit, which lies between 14 and 22 at.%. Above that limit the proliferation of cells decreases despite the increase in the surface wettability. This indicates that the biological response is not determined by the surface wettability alone. A balance must be maintained between the polar and dispersive components of the SFE, so that the extracellular matrix proteins are firstly attached to the surface. Otherwise, the preferential attachment of water to the surface may occur and limit the adhesion of cells.

Further research in order to precisely determine the optimal content of Si for cells is required and planned by the authors. An in-depth study of the interactions of cells and biomaterials’ surface will allow a better understanding of the mechanisms underlying the cells’ adhesion and proliferation.

## Figures and Tables

**Table 1:** Chemical composition of the deposited DLC and Si-DLC coatings according to the XPS analysis.

Sample	Si Target Power [W]	Si [at.%]	O [at.%]	C [at.%]	N [at.%]
DLC	0	-	9.84 ± 0.11	89.48 ± 0.24	0.68 ± 0.21
Si-DLC 0	10	1.83 ± 0.01	13.72 ± 0.16	84.45 ± 0.41	-
Si-DLC 1	20	3.79 ± 0.04	21.93 ± 0.34	74.28 ± 0.30	-
Si-DLC 2	40	5.58 ± 0.03	18.80 ± 0.07	75.62 ± 0.10	-
Si-DLC 3	60	14.34 ± 0.10	28.90 ± 0.30	56.76 ± 0.19	-
Si-DLC 4	80	22.15 ± 0.37	24.85 ± 0.18	53.00 ± 0.54	-

**Table 2:** Decomposition of the C 1s peak for the examined DLC and Si-DLC coatings.

Sample	Si-C/SiO_x_C_y_	C sp^2^	C sp^3^	C-O	C=O	COO-R	sp^2^/sp^3^
DLC	-	59.28 ± 0.69	17.98 ± 0.65	11.86 ± 0.07	5.95 ± 0.04	4.93 ± 0.01	3.30 ± 0.16
Si-DLC 0	1.50 ± 0.00	71.70 ± 1.00	13.50 ± 0.10	7.80 ± 0.20	3.65 ± 0.25	2.10 ± 0.50	5.30 ± 0.11
Si-DLC 1	3.30 ± 0.10	63.95 ± 0.75	14.85 ± 0.05	9.50 ± 0.20	4.70 ± 0.20	3.70 ± 0.20	4.31 ± 0.07
Si-DLC 2	9.30 ± 1.60	57.15 ± 4.65	16.95 ± 2.95	8.95 ± 0.15	1.70 ± 0.20	5.50 ± 0.30	3.53 ± 0.89
Si-DLC 3	18.10 ± 0.40	53.55 ± 0.65	14.30 ± 0.80	7.45 ± 0.25	3.65 ± 0.05	2.95 ± 0.05	3.76 ± 0.26
Si-DLC 4	36.00 ± 0.50	42.85 ± 0.75	13.65 ± 0.05	4.70 ± 0.20	1.35 ± 0.05	0.95 ± 0.05	3.14 ± 0.07

**Table 3:** Decomposition of the O 1s peak for the obtained Si-DLC coatings.

Sample	C=O	Si-O-C/SiO_x_C_y_	SiO_x_
Si-DLC 0	11.85 ± 0.95	67.45 ± 1.35	20.70 ± 2.30
Si-DLC 1	7.20 ± 1.10	69.25 ± 1.05	23.55 ± 2.15
Si-DLC 2	7.15 ± 2.25	70.15 ± 0.15	22.70 ± 2.10
Si-DLC 3	1.25 ± 0.25	83.15 ± 0.25	15.60 ± 0.20
Si-DLC 4	-	84.85 ± 0.15	15.15 ± 0.15
